# Degradome Analysis of Tomato and *Nicotiana benthamiana* Plants Infected with Potato Spindle Tuber Viroid

**DOI:** 10.3390/ijms22073725

**Published:** 2021-04-02

**Authors:** Beatriz Navarro, Andreas Gisel, Pedro Serra, Michela Chiumenti, Francesco Di Serio, Ricardo Flores

**Affiliations:** 1 Istituto per la Protezione Sostenibile delle Piante, Consiglio Nazionale delle Ricerche, 70126 Bari, Italy; beatriz.navarro@ipsp.cnr.it (B.N.); michela.chiumenti@ipsp.cnr.it (M.C.); 2 Istituto di Tecnologie Biomediche, Consiglio Nazionale delle Ricerche, 70126 Bari, Italy; A.Gisel@cgiar.org; 3 International Institute of Tropical Agriculture, Ibadan 200001, Nigeria; 4 Istituto de Biología Molecular y Celular de Plantas (CSIC-UPV), 46022 Valencia, Spain; pedseral@alumni.upv.es (P.S.); rflores@ibmcp.upv.es (R.F.)

**Keywords:** RNA silencing, viroid-derived small RNAs, PARE analyses, miRNA, pathogenesis

## Abstract

Viroids are infectious non-coding RNAs that infect plants. During infection, viroid RNAs are targeted by Dicer-like proteins, generating viroid-derived small RNAs (vd-sRNAs) that can guide the sequence specific cleavage of cognate host mRNAs via an RNA silencing mechanism. To assess the involvement of these pathways in pathogenesis associated with nuclear-replicating viroids, high-throughput sequencing of sRNAs and degradome analysis were carried out on tomato and *Nicotiana benthamiana* plants infected by potato spindle tuber viroid (PSTVd). Both hosts develop similar stunting and leaf curling symptoms when infected by PSTVd, thus allowing comparative analyses. About one hundred tomato mRNAs potentially targeted for degradation by vd-sRNAs were initially identified. However, data from biological replicates and comparisons between mock and infected samples reduced the number of bona fide targets—i.e., those identified with high confidence in two infected biological replicates but not in the mock controls—to only eight mRNAs that encode proteins involved in development, transcription or defense. Somewhat surprisingly, results of RT-qPCR assays revealed that the accumulation of only four of these mRNAs was inhibited in the PSTVd-infected tomato. When these analyses were extended to mock inoculated and PSTVd-infected *N. benthamiana* plants, a completely different set of potential mRNA targets was identified. The failure to identify homologous mRNA(s) targeted by PSTVd-sRNA suggests that different pathways could be involved in the elicitation of similar symptoms in these two species. Moreover, no significant modifications in the accumulation of miRNAs and in the cleavage of their targeted mRNAs were detected in the infected tomato plants with respect to the mock controls. Taken together, these data suggest that stunting and leaf curling symptoms induced by PSTVd are elicited by a complex plant response involving multiple mechanisms, with RNA silencing being only one of the possible components.

## 1. Introduction

Viroids are non-protein coding RNAs that infect plants [1]. According to their structural, functional and biological properties, viroids have been classified in the families *Pospiviroidae* (type member, potato spindle tuber viroid, PSTVd) and *Avsunviroidae* (type member, avocado sunblotch viroid, ASBVd) that group nuclear and chloroplast replicating viroids, respectively [2,3]. Members of both families may cause plant diseases, but how viroid RNAs may elicit pathogenesis is a question only partially answered [4].

Both nuclear and chloroplast replicating viroids are triggers and targets of RNA silencing, a regulatory mechanism of gene expression in most eukaryotes [5]. RNA silencing is mediated by microRNAs (miRNAs) and small interfering RNAs (siRNAs), which are small RNAs (sRNAs) of 21–24 nt originated by type III RNases (Dicer-like, DCLs in plants) targeting for degradation highly structured or double-stranded (ds) RNAs, respectively [6]. After loading into Argonaute proteins (AGOs), which are part of the RNA inducing silencing complex (RISC), siRNAs and miRNAs direct such complexes to either cognate mRNAs for their specific cleavage or translation inhibition or to cognate DNA where they guide RNA-dependent DNA methylation [7]. The identification of viroid-derived small RNAs (vd-sRNAs) resembling miRNAs and siRNAs in viroid infected tissues [8,9,10] prompted the proposal that vd-sRNAs, besides being involved in antiviroid defense [reviewed in 4], could target for degradation certain cellular mRNAs encoding protein(s) essential for a relevant host molecular pathway(s), thus causing macroscopic symptoms [9,11]. Immunoprecipitation assays showed that vd-sRNAs are incorporated into AGO proteins in vivo [12] according with the size and 5′ terminal nucleotide constraints operating on miRNAs and siRNAs [13,14].

Evidence that vd-sRNAs may actually be the initial molecular event of macroscopic symptoms were obtained for sequence variants of peach latent mosaic viroid (PLMVd) [15] causing a severe albinism (peach calico, PC) or a yellow mosaic (peach yellow mosaic, PYM). The pathogenic determinants of the two diseases were mapped at different sites in the PLMVd-PC and PLMVd-PYM variants [16,17,18]. It was shown that vd-sRNAs containing the PC or the PYM pathogenic determinant targeted for degradation the nuclear-encoded chloroplastic HSP90 (cHSP90) or the thylakoid translocase subunit (cpSecA) in PC and PYM, respectively [18,19], two proteins involved in chloroplast development. Therefore, in both cases, a short signaling cascade, linking the impairment of the expression of a single protein with the macroscopic symptom, appeared to be activated by different PLMVd-sRNAs. The observed phenotypes in the PC- and PYM-affected plants were different from each other, in agreement with the impairment of different proteins interfering with different chloroplast developmental pathways [4].

The role of vd-sRNAs in pathogenesis of nuclear replicating viroids has been studied in several viroid-host combinations [5]. When stunting and leaf curling symptoms induced by several variants of PSTVd in tomato were examined, mRNAs encoding two callose-like synthase, a chloride channel protein, the ribosomal protein S3a, two receptor-like serine/threonine-protein kinase, a serine/threonine-protein kinase, the phosphatidylinositol 4-kinase alpha protein, the vacuole membrane protein 1 and a pentatricopeptide repeat-containing protein were identified as targets or potential targets of specific PSTVd-sRNAs [20,21,22]. Impaired expression of these genes was associated with the typical stunting and leaf curling symptoms induced by PSTVd. These studies examined only one or a few potential targets, thus leaving open the question of their simultaneous involvement in pathogenesis. Data providing a global view of the mRNAs targeted for degradation by PSTVd-sRNAs in the infected tissues are so far limited to a single study in tomato [23]. Moreover, comparative analysis of the mRNAs targeted by the same viroid in different hosts is missing.

A global view of the mRNAs targeted for degradation by RNA silencing can be achieved by degradome sequencing, a genome-wide approach that allows the identification of most uncapped 5′ phosphate termini of mRNAs in a sample, including those generated by the sequence-specific AGO1-endonucleotidic cleavage guided by miRNA/siRNAs [24,25]. To examine the role of vd-sRNAs in PSTVd/tomato interaction in greater detail, we applied this wide-genome experimental approach associated with high-throughput sequencing (HTS) of small RNAs to PSTVd-infected and non-infected tomato plants. We also performed comparative degradome analysis in tomato and *N. benthamiana*, two plant species that respond to PSTVd infection by developing similar stunting and leaf curling symptoms.

## 2. Results

### 2.1. Analyses of Viroid-Derived Small RNAs in Tomato and N. benthamiana

**Figure 1:**
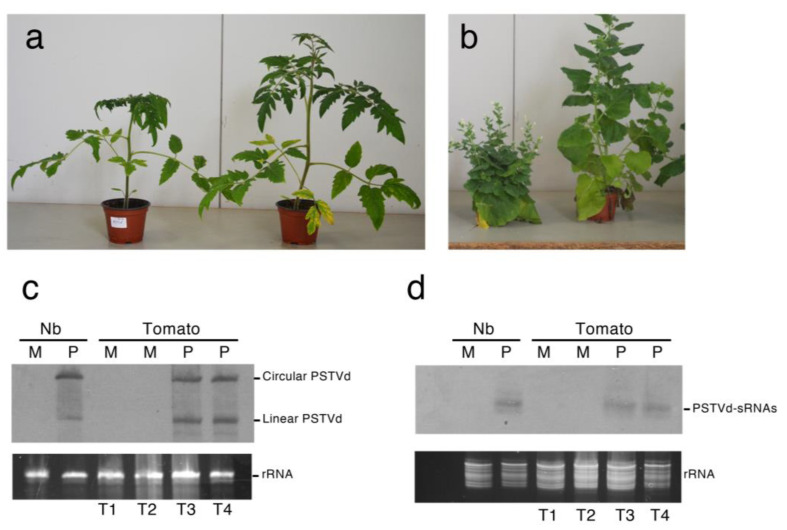
Symptoms caused by PSTVd-Nb infections in tomato (**a**) and *N. benthamiana* (**b**). PSTVd-infected plants of each species showing typical symptoms of stunting and leaf curling at 30 dpi (left) are compared with the respective mock-inoculated plants (right). (**c**,**d**) accumulation of circular and linear genomic PSTVd RNAs (**c**) and PSTVd-derived small RNAs (PSTVd-sRNAs) (**d**) at 30 dpi in *N. benthamiana* and tomato plants. The total RNA preparations from mock-inoculated (M) and PSTVd-infected (P) tomato and *N. benthamiana* (Nb) plants were separated by denaturing PAGE in 5% (**c**) or 17% (**d**) gels and analyzed by Northern-blot hybridization with a DIG-labeled riboprobe for detecting PSTVd (+) strand. Equal loading was assessed by the intensity of the bands corresponding to rRNAs after staining with ethidium bromide (**c**,**d**, bottom).

Tomato and *N. benthamiana* seedlings inoculated with the PSTVd variant Nb were analyzed 30 days post-inoculation (dpi), when typical symptoms of stunting and leaf curling were clearly observed (Figure 1a,b). At this time point, two mock (T1 and T2) and two PSTVd-inoculated tomato plants (T3 and T4) and one mock- and one PSTVd-inoculated *N. benthamiana* plant were selected for further analyses. First, PSTVd infection and accumulation of PSTVd-sRNAs in the inoculated plants were confirmed by Northern-blot hybridization with an RNA probe complementary to (+) genomic RNA (Figure 1c,d).

These assays revealed that the circular and linear forms of PSTVd and the (+) PSTVd-sRNAs accumulated at similar levels in both inoculated tomato replicates. The same RNA preparations were then used to generate six libraries of sRNAs and six libraries of degradome RNAs corresponding to the two mock and two infected tomato biological replicates and to the infected and mock *N. benthamiana* plants. Sequencing of each sRNA library yielded a total of 39–51 million high quality reads 18–26 nt long. Up to 14–17% of the total sRNAs reads from the infected samples were derived from PSTVd, with very similar levels of PSTVd-sRNAs (about 6.3–7.3 million) in the three libraries. In agreement with previous studies [20,26,27], PSTVd-sRNAs of both polarities, with a predominance of those of plus polarity, were identified (Figure S1). When (+) and (−) PSTVd-sRNAs from the two tomato replicates and one *N. benthamiana* plant infected by PSTVd were mapped on PSTVd genomic and complementary RNAs, an irregular distribution of the reads was found, with very similar profiles observed for all three libraries (Figure S2). These findings suggest that the observed read distributions may be host species-independent.

### 2.2. Identification of Tomato mRNAs Cleaved by miRNA via RNA Silencing

Between 38–57 million clean tags, representing the 5′ ends of uncapped, polyadenylated RNAs, were obtained in the degradome libraries prepared from the two mock- and two PSTVd-inoculated tomato replicates. While tags resulting from the cleavage of mRNAs directed by host miRNAs and siRNAs were expected in all samples, the PSTVd-infected replicates should also contain tags corresponding to host mRNAs specifically targeted by PSTVd-sRNAs. In addition, because degradome analysis also identifies random degradation products that are not generated by endonucleolytic cleavage mediated by RNA silencing machinery [28], false positives were also expected. To exclude as many as possible of these false positive targets of PSTVd-sRNAs, we adopted several controls and stringent screening conditions. Putative cleaved targets identified by PAREsnip [28] were classified into five categories (i.e., categories 0–4) according to the abundance of degradome tags at the cleaved position. This classification indicates the confidence level of the sRNA-mediated cleavages, those in category 0 being the most certain and those in categories 3 and 4 the most uncertain, likely corresponding to background noise generated by random degradation.

As a preliminary test of the quality of the degradome sequencing results, we used the PAREsnip software to identify in the four degradome libraries those mRNAs that were cleaved by the conserved tomato miRNAs. A total of 84 mRNAs were identified as targets of miRNAs belonging to 24 different miRNA families (Table S1), and most (75%) miRNA/mRNA pairs were classified as category 0 or 1 that, according to PAREsnip categorization, provide the strongest empirical evidence for a true cleavage at the specific position. This result was highly reproducible among the four libraries. Most of the miRNA targets identified in this study have been previously reported [23,29,30,31,32,33]. However, 14 appeared to be new potential targets of previously reported miRNAs (Table S1).

Target plots (t-plots) were generated for several selected tomato mRNA targets of miRNAs, including miR-156, miR-167, miR-169, miR-172, mi-R319, miR-396, miR-398, and miR-408 to verify the quality of the sequenced libraries (Figure S3). Similar low levels of background degradation were observed for each transcript in libraries from mock- and PSTVd-inoculated samples (Figure S3), highlighting the reproducibility of the degradome analyses and the consistency of our results with those from other studies [23,29,30,31,32,33]. When data from the mock and PSTVd-infected samples were compared, changes in the abundance of degradome tags of the targeted mRNAs were not significant (*p* < 0.05, Table S1) suggesting that miRNA-mediated cleavages remained largely unaffected upon PSTVd infection. Based on data from the corresponding sRNA libraries, levels of all but four miRNAs (i.e., miR169e-3p, miR169-5p, miR-171, and miR-4376) were also not significantly affected upon PSTVd infection (Figure S4). Notably, changes in the abundance of these four miRNAs were not associated with a significant parallel change in the cleavage of the specific targets (Table S1). Altogether, these data, besides suggesting that PSTVd infection did not significantly affect the cleavage activity mediated by miRNAs in tomato, provided evidence of the high quality and repeatability of the degradome data from tomato plants.

### 2.3. Identification of Tomato mRNAs Cleaved by PSTVd-sRNAs via RNA Silencing

**Figure 2:**
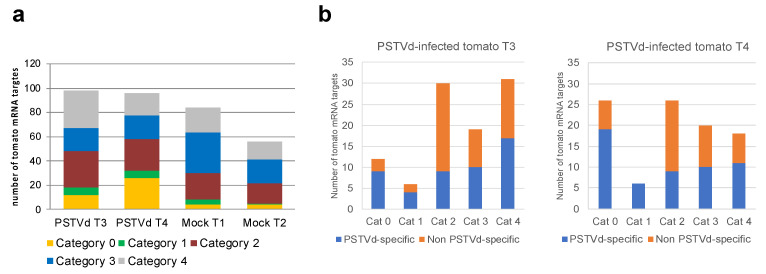
(**a**) Total number and quality categorization (from 0 to 4, where 0 represents the strongest evidence for true cleavage products) of mRNAs targeted by PSTVd-sRNAs in PSTVd-infected (T3 and T4) and of non-PSTVd specific (false positive) mRNAs targets in mock-inoculated (T1 and T2) tomato biological replicates identified by degradome analysis; (**b**) number of PSTVd specific and non-specific mRNA targets in each viroid-infected tomato plant (T3 and T4) and degradome category. The PSTVd-specific mRNA targets were those not identified in any of the two mock-inoculated tomato replicates (T1 and T2), which are all considered as false positives.

The mRNAs potentially targeted by PSTVd-sRNAs in the viroid-infected tomato plants were identified using the PAREsnip software and a subset of 21-nt PSTVd-sRNAs extracted from the respective sequenced sRNA libraries. Almost 100 potentially targeted mRNAs were identified in each library from infected samples (Tables S2 and S3). These mRNAs contained potential cleavage sites belonging to all categories (from 0 to 4), with category 0 sites being 17–22% of the total identified targets (Figure 2a). Surprisingly, 60–80 possible mRNA targets were also identified in the two degradome libraries from the mock-inoculated tomato plants, when PAREsnip was applied using all 21-nt PSTVd sRNAs potentially generated by the variant PSTVd-Nb (Tables S4 and S5). In this case, the cleavage sites of the identified mRNAs also belonged to all PAREsnip categories, but targets with cleavage sites of category 0 were significantly less abundant (2–3%) than in the PSTVd-infected samples (Figure 2a). Since mock-inoculated plants did not contain PSTVd-sRNAs, all the potential targets identified in these samples must correspond to degradation products not generated by vd-sRNA-directed endonucleolytic cleavages, and therefore can be considered as non-PSTVd specific (i.e., false positive). When these false targets were removed from the list of potential targets identified in the two PSTVd-infected libraries, a total of 49 and 56 targets of PSTVd-sRNAs belonging to all four categories were left in the T3 and T4 libraries, respectively (Figure 2b), hereafter denoted as PSTVd specific targets.

**Figure 3:**
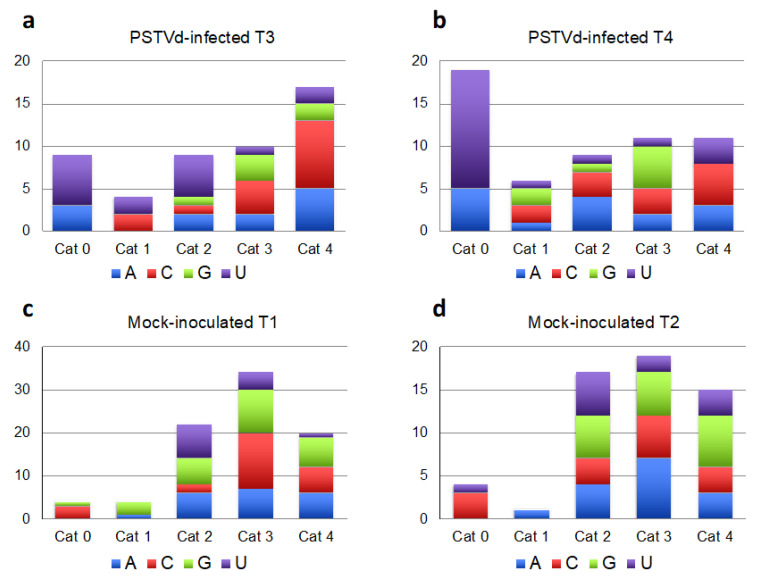
Analysis of the 5′ terminal nucleotide of PSTVd-sRNAs involved in the cleavage of PSTVd-specific targets according with the degradome category (**a**) and (**b**). In PSTVd-infected tomato samples T3 (**a**) and T4 (**b**), the 5′ terminal nucleotide of PSTVd-sRNAs involved in host mRNA cleavages classified into the category 0 were U or A, supporting their incorporation into AGO1. When the same analysis was performed considering the false targets identified in the degradome sequencing from mock-inoculated samples (T1 and T2, (**c**) and (**d**), respectively), such a bias was not observed.

Taking into consideration the fact that vd-sRNAs targeting host mRNAs are expected to be incorporated into AGO1 and that the nucleotide at the 5′ end of the vd-sRNA contributes to its loading into AGO1 [12], we examined the 5′ terminus of each of the respective PSTVd-sRNA. Interestingly, vd-sRNAs involved in cleavage of PSTVd specific targets falling into category 0 had exclusively U or A at their 5′ terminus, as expected for AGO1 loading (Figure 3a,b). In contrast, a similar bias was not observed at the 5′ nt of the vd-sRNAs from the other categories or for the vd-sRNAs targeting mRNAs classified as probable false positives in the degradome from mock-inoculated controls (see above, Figure 3c,d).

These data indicate that the chance of identifying false targets of PSTVd-sRNAs in the degradome libraries from infected samples is lower when the cleavage sites fall within the category 0. In addition, since the two biological replicates showed identical symptoms, we reasoned that key mRNA(s) targeted by vd-sRNAs and involved in pathogenesis should be found in both replicates. Consequently, category 0 mRNAs that were present in both replicates after the exclusion of potential false positives were further considered as bona fide targets of PSTVd-sRNAs. These correspond to eight tomato mRNAs targeted by eight different PSTVd-sRNAs derived mainly from the plus polarity strand, with only two of them being of negative polarity (Table 1). Irrespective of polarity, all PSTVd-sRNAs mediating the cleavage of the bona fide targets originated outside the hot-spots observed in the distribution profile of PSTVd-sRNAs on the viroid RNA (Figure S2), indicating that sRNA abundance may not be directly correlated with mRNA targeting. Interestingly, these PSTVd-sRNAs are derived from sequence regions that are conserved among several PSTVd strains showing different pathogenicity; e.g., the intermediate, mild, RG1 and lethal variants (Table 1 and Figure S5). Only two of them, with 5′ ends mapping at positions 192 and 290, are not conserved in the mild variant (Table 1).

The bona fide targeted mRNAs encode eight proteins annotated as CWF19 (CWF19, Solyc09g083420.2.1), Little nuclei 1 (LINC1, Solyc03g045050.2.1), subunit of origin recognition complex protein 6 (ORC6, Solyc05g007450.2.1), NAC domain containing protein 38 (NAC038, Solyc04g079940.2.1), Supercentipede 1 (SCN1, Solyc11g012270.1.1), cyclin-dependent serine/threonine kinase F-1 (CDKF-1, Solyc12g007200.1.1), putative serine-threonine kinase (PBS1, Solyc05g024290.2.1) and proton pump interactor 1 (PPI-1, Solyc07g063100.2.1) (Table 1). Four of these proteins (CWF19, NAC38, LINC1 and ORC6) are localized in the nucleus, two of them being transcription factors. CWF19 belongs to the CWFJ-like family protein of transcription factors containing a CCCH-type zinc finger and is likely involved in mRNA splicing, as denoted by its GO classification. NAC38 is a member of the NAC-family of plant transcription factors involved in developmental processes as well as in the responses to abiotic and biotic stresses, plant hormonal control, and defense [34,35]. In Arabidopsis, *LINC1* encodes a coiled-coil nuclear protein that acts as a positive regulator of pathogen-associated molecular pattern-triggered immunity (PTI) responses, thereby regulating jasmonic acid signaling and gibberellin biosynthesis [36]. This protein also contributes to the shape and size of the nucleus [37]. *ORC6* is one of the components required for assembly of the pre-replication complex necessary to initiate DNA replication in Arabidopsis [38,39], and it is involved in the BRP4-mediated mitotic cell-cycle progression [40]. The other four targeted mRNAs (*SCN1*, *CDKF1*, *PBS-1*, and *PPI-1*) correspond to genes encoding proteins that are not localized in the nucleus. *SCN1* encodes a RhoGTPase GDP dissociation inhibitor (RhoGDI), which regulates root hair growth in Arabidopsis [41]. *CDKF-1* encodes a cyclin-dependent serine/threonine protein kinase that is involved in activating phosphorylation of cyclin dependent kinases in Arabidopsis [42] and is a major regulator of cell proliferation and cell expansion [43]. Interestingly, Arabidopsis cdkf;1-1 mutants exhibited defects in cell division and cell elongation, leading to severe growth retardation in both shoots and roots [43]. In Arabidopsis, the serine-threonine kinase PBS1 is involved in plant defense against bacteria [44]. *Arabidopsis thaliana* PPI1 homolog protein is involved in proton transport through the plasma membrane [45,46].

### 2.4. Evidence for Cleavage of the Bona Fide Targets by PSTVd-sRNAs

**Figure 4:**
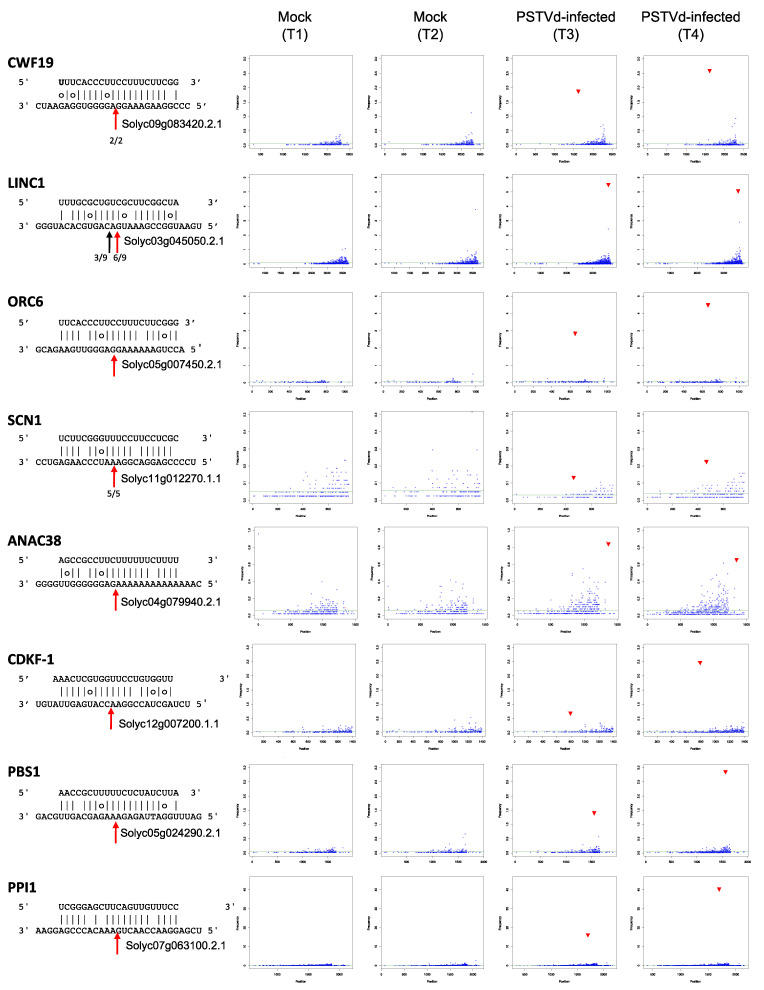
T-plots of eight bona fide mRNA targets of PSTVd-sRNAs. The abundance of each degradome tag is plotted as a function of the position of its 5′ end in the transcript. In each t-plot, the tag abundance at the cleavage site according with the cognate PSTVd-sRNA is reported in red. The duplex PSTVd-sRNA:mRNA target is shown on the left, with the cleavage site position indicated by a red arrow and the number of RLM–RACE clones validating the cleavage site indicated (in CWF19, LINC1, SCN1).

Analyses of cleavages throughout the sequence of the eight bona fide target mRNAs (t-plot analyses) showed that, in the PSTVd-infected samples, the frequency of 5′ ends consistent with the specific PSTVd-sRNA-mediated cleavage was always higher than the respective degradation background, with similar results obtained for both biological replicates (Figure 4, Table 1). A comparable degradation background was observed in the t-plots corresponding to the same mRNAs from mock-inoculated samples where, as expected, mRNAs with 5′ ends consistent with PSTVd-sRNAs-mediated cleavages were not found (Figure 4). Therefore, in contrast to previous reports regarding other PSTVd-sRNA-targeted tomato mRNAs coding for CLC-b-like and RPS3a-like proteins [21], cleavage of the bona fide target mRNAs identified here is not associated with a vd-sRNA-independent degradation induced by viroid infection at late stages. To further validate these results, the PSTVd-sRNAs cleavage sites of three of the identified bona fide targets (Solyc09g083420.2.1, Solyc03g045050.2.1 and Solyc11g012270.1.1 encoding CWF19, LINC1 and SCN1, respectively) were determined by 5′ RLM-RACE. All cDNA clones derived from Solyc09g083420.2.1 and Solyc11g012270.1.1 mRNA fragments had 5′ termini compatible with a cleavage mediated by the respective targeting PSTVd-sRNAs (Figure 4); In the case of Solyc03g045050.2.1, six out of nine clones had cDNAs with 5′ termini corresponding to the predicted cleavage site; While, in three clones, the termini mapped one nucleotide downstream in the mRNA (Figure 4). Taken together, these data confirmed the cleavage of targeted mRNAs according to an RNA silencing mediated mechanism guided by the respective PSTVd-sRNAs.

### 2.5. Correlation between Targeting by PSTVd-sRNAs and Steady State Level of Cleaved Host mRNAs

**Figure 5:**
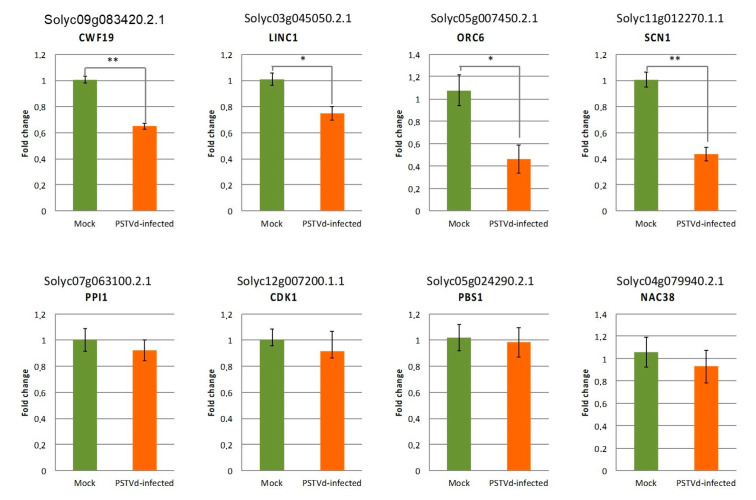
Accumulation levels of the eight bona fide mRNA targets of PSTVd-sRNAs in PSTVd-infected with respect to mock-inoculated tomato plants determined by RT-qPCR. The reported data are the mean of at least three independent experiments. Error bars indicate standard errors; Statistical significance for the *t*-test is indicated by asterisks: * *p* < 0.005; ** *p* < 0.001.

The effect of the mRNA silencing-mediated cleavage on the steady state levels of the eight bona fide targets of PSTVd-sRNAs was tested by RT-qPCR assays on three biological replicates from three independent inoculation experiments. To amplify cDNAs derived exclusively from the uncleaved mRNAs, specific PCR primers were designed to bind upstream and downstream from the cleavage site of each target. Only the mRNAs encoding CWF19, LINC1, ORC6, and SCN1 showed a significantly lower level of accumulation in the PSTVd-infected than in the mock-inoculated tomato plants (Figure 5), supporting a role for the respective PSTVd-sRNAs in the regulation of the expression of the cognate genes. In contrast, no significant difference was observed in the accumulation of the other four bona fide targets, indicating that cleavage induced by the specific PSTVd-sRNAs is not always followed by significant changes in the steady state level of the cleaved mRNAs (Figure 5).

### 2.6. Identification of N. benthamiana mRNAs Potentially Cleaved by miRNAs and PSTVd-sRNAs via RNA Silencing

Sequencing of degradome libraries of mock and PSTVd-infected *N. benthamiana* plants generated 23 and 28 million of clean reads, respectively. PAREsnip analysis identified a total of 79 mRNAs cleaved by conserved miRNAs belonging to 15 different families. Approximately 70% of the cleavage sites in both the mock-inoculated and PSTVd-infected samples were classified as category 0 or 1 according to the PAREsnip categorization (Table S6). This high degree of specificity was consistent with data reported in the literature [47], thus validating the results of this degradome analysis.

**Figure 6:**
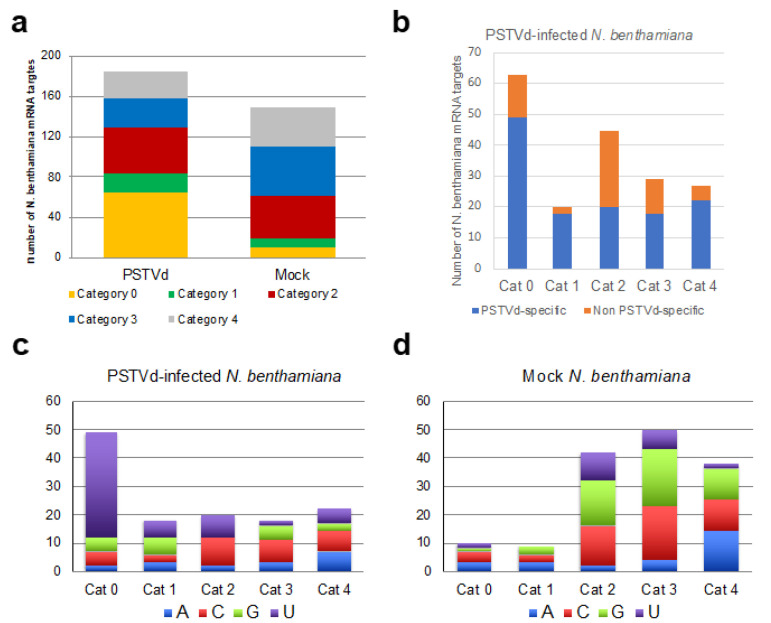
(**a**) Total number and quality categorization (from 0 to 4, where 0 represents the strongest evidence for true cleavage products) of mRNA targets of PSTVd-sRNA and non-PSTVd specific (false positive) mRNAs targets identified by degradome analysis in PSTVd-infected and mock-inoculated *N. benthamiana*, respectively; (**b**) number of specific and non- PSTVd specific mRNA targets in PSTVd-infected *N. benthamiana* plant in each degradome category. The PSTVd-specific mRNA targets were those not identified in any of the two mock-inoculated tomato replicates; (**c**) analysis of the 5′ terminal nucleotide of PSTVd-sRNAs involved in the cleavage of PSTVd-specific targets according to the degradome category in PSTVd-infected *N. benthamiana*, the 5′ terminal nucleotide of PSTVd-sRNAs involved in host mRNA cleavages classified into the category 0 were mainly U. (**d**) When the same analysis was performed considering the PSTVd-sRNAs targeting the false (non-PSTVd-specific) targets identified in the degradome sequencing from mock-inoculated samples, no bias was observed in the 5′ terminal nucleotide of PSTVd-sRNAs targeting mRNAs of category 0, suggesting that they were not targeted by AGO1.

Paralleling the search strategy adopted for tomato, a total of 184 or 149 potential PSTVd-sRNA targets belonging to all PAREsnip categories were identified in the RNA samples isolated from PSTVd-infected and mock-inoculated *N. benthamiana*, respectively (Tables S7 and S8). Nearly half of the targets identified in the PSTVd-infected plant belonged to category 0 (Figure 6a). Subtraction of false positive targets identified in the mock-inoculated plant from the potential targets identified in the PSTVd-infected library yielded a total of 49 targets of category 0 (Figure 6b and Table S9). As observed for tomato, most (a total of 37) mRNAs containing category 0 cleavage sites were targeted by PSTVd-sRNAs with a U at their 5′ terminus (Figure 6c), supporting their preferential loading into AGO1. A similar bias was not observed for either vd-sRNAs in the categories 1–4 or those in category 0 identified in the mock-inoculated controls (false positive targets, Figure 6d). t-plots of representative targets of PSTVd-sRNAs in the mock and PSTVd-infected samples showed comparable degradation backgrounds (Figure S6). Therefore, as observed in tomato, PSTVd-infection in *N. benthamiana* was not accompanied by a non-specific degradation of mRNAs targeted by PSTVd-sRNA. In the absence of a replicate, it was not possible to determine which were bona fide targets of PSTVd-sRNAs in *N. benthamiana*; thus, some of the potential targets could be false. It should be noted, however, that none of the 49 mRNAs potentially targeted by PSTVd-sRNAs in *N. benthamiana* (Table S5) corresponded to bona fide or potential targets identified in tomato infected by the same strain of PSTVd and showing similar symptoms. Moreover, although the PSTVd-sRNAs mediating the cleavage of the bona fide targets in tomato were also identified in *N. benthamiana*, they are not expected to target the *N. benthamiana* homologs because nucleotide changes in the mRNA sequence would destabilize the corresponding vd-sRNA/mRNA duplexes and impair their AGO-mediated cleavage (Table S10).

## 3. Discussion

Sequence-specific cleavage of host mRNAs mediated by vd-sRNAs via RNA silencing has been proposed to be one of the primary molecular lesions that could initiate a cascade of molecular events leading to the development of macroscopic symptoms in viroid infected plants. To assess the potential involvement of this mechanism in the pathogenesis of PSTVd, the type member of the family of nuclear-replicating viroids, we performed comparative analyses of sRNAs and degradome libraries from tomato and *N. benthamiana* plants either mock-inoculated or infected by the same PSTVd-Nb variant. These two solanaceous species were selected because they develop similar stunting and leaf curling symptoms when infected by PSTVd-Nb, thus providing the possibility of determining whether or not homologous genes or genes involved in the same developmental pathway were impaired by RNA silencing activated by PSTVd-sRNAs. A genome-wide analysis strategy based on HTS of sRNAs and degradome libraries was adopted to gain a global view of the host mRNAs targeted by vd-sRNAs in the two hosts.

When the degradome libraries were screened for mRNAs targeted by conserved miRNAs, previously reported cleavages of tomato mRNAs by conserved miRNAs were detected [23,29,30,31,32,33]. Moreover, several new targets were identified, thereby confirming the depth of the degradome sequencing. Comparisons of sRNA data from the mock-inoculated and PSTVd-infected tomato replicates showed that most miRNAs accumulated to similar levels, with significant alterations observed for only four miRNAs. These changes were not associated with significant parallel quantitative changes in the cleavage of the respective targeted mRNAs, however. These data were consistent with those previously reported by Zheng and colleagues [23], who found that increased levels of two of these miRNAs (miR-171 and miR-4376) following PSTVd-infection were not accompanied by significant changes in the cleavage and accumulation of the respective targeted transcripts. Nevertheless, we did not observe significant alterations in the miR-167- and miR-393-guided cleavage of Solyc02g037530 (encoding auxin response factor 8) and Solyc09g074520 (encoding TIR1), respectively, as reported by the same authors.

Based on HTS data, alterations in the accumulation levels of some miRNAs have previously been reported in tomato plants infected by several PSTVd variants [48,49]. A third study by Owens and colleagues reported that miRNA levels were either unchanged or only moderately affected in PSTVd-infected plants [50]. Interestingly, no significant differences in the accumulation levels of any of the miRNAs examined or their respective mRNA targets were detected in tomato plants infected by citrus exocortis viroid, a viroid closely related to PSTVd [51]. This apparent contradiction calls for additional studies to conclusively assess the involvement of miRNAs in plant–viroid interaction.

To screen out potential false targets in the degradome libraries resulting from random degradation products, our search for tomato mRNAs targeted by PSTVd-sRNAs was carried out adopting stringent criteria that took into consideration both false positives identified in the degradome library from mock-inoculated plants and the statistical robustness of the mRNA cleavage. Moreover, to increase the chances of pinpointing real targets, only those targets identified in both degradome libraries from symptomatic plants were considered. Applying this approach, the almost 100 potential targets predicted based on degradome analysis in the absence of constraints were reduced to eight bona fide mRNA targets of PSTVd-sRNAs. All the PSTVd-sRNAs mediating the cleavage of the bona fide targets have either a U or an A nucleotide at the 5′ end, thus satisfying the preferences for loading into AGO1 [12,13,14], the key enzyme operating in RNA silencing against endogenous and foreign RNAs [52,53,54]. Interestingly, these PSTVd-sRNAs accumulate to only relatively low levels (Table 1) and did not correspond to those forming the hot-spots observed in the distribution profiles of both PSTVd plus and minus RNAs (Figure S2). A previous report noted the relatively low abundance of PSTVd-sRNAs involved in the silencing of endogenous tomato mRNAs [21], paralleling the situation observed for the chloroplast replicating viroid PLMVd [19]. Taken together, these data indicate that the biological significance of vd-sRNAs in silencing host mRNAs cannot be inferred solely on the basis of their relative abundance in the infected tissues.

The eight bona fide mRNAs targeted by PSTVd-sRNAs encode proteins having potential roles in relevant developmental, signaling, or defense pathways. These proteins are localized in either the nucleus or cytoplasm. In our study, the t-plots and RACE analysis of infected samples confirmed that the bona fide targets were predominantly cleaved at the predicted sites. Nevertheless, RT-qPCR assays showed that only four of the eight target mRNAs accumulated at significantly lower levels in the viroid-infected hosts than in the mock-inoculated plants. Therefore, the effective cleavage mediated by PSTVd-sRNAs did not produce significant changes in the steady-state level of at least some targeted mRNAs.

This observation is in agreement with results of a previous degradome analyses of PSTVd-infected tomato plants [23], although the targeted mRNAs in the latter study were different from those reported here. It is possible that a temporary transcriptional induction of the targeted mRNAs may compensate for the reduction due to cleavage. In our study, the targeted mRNAs whose accumulation remained apparently unchanged in the infected tissues encode either signaling proteins such as protein kinases CDKF-1 and PBS1, or proteins such as CDKF1 and NAC38 that are involved in specific developmental or defense pathways. Rather than being constitutively expressed, expression of such proteins would be expected to be induced during the viroid infection. Therefore, we cannot exclude the possibility that the basal PSTVd-sRNA-mediated cleavage of the cognate mRNAs may became physiologically relevant during specific plant developmental stages, especially if the transcription of the targeted mRNAs is simultaneously affected by other regulatory networks in infected tissues.

Among the mRNAs targeted by PSTVd-sRNAs and exhibiting a significantly lower steady state level in the infected tissues were those encoding nuclear proteins CWF19, which is likely involved in mRNA splicing, and the homolog of the Arabidopsis SCN1 protein, involved in root hair growth. Previous reports have documented both alterations in the alternative splicing of host protein-coding genes [23] and downregulation of the *SCN1* gene [55] in PSTVd-infected tomato plants.

The fact that all the PSTVd-sRNAs targeting the bona fide mRNAs identified in this study derived from regions of the genome that are conserved among different PSTVd strains suggests that cleavage of some of the targeted mRNAs could be relevant for viroid replication and/or infectivity. Further studies are needed to explore this possibility. However, because different PSTVd strains induce symptoms of differing severity, a direct link between these eight particular PSTVd-sRNAs and viroid pathogenicity seems unlikely.

None of the tomato mRNAs identified as targets for degradation by PSTVd-sRNAs in previous studies [20,21,22,23,56] were detected in our degradome libraries, even when searches were extended to targets belonging to all categories or those not present in both replicates. Differences in PSTVd strains, tomato varieties, and the experimental conditions used in each study could explain these discrepancies. Taken together, these data indicate that (i) PSTVd-sRNAs may simultaneously target different mRNAs for sequence-specific cleavage in infected tomato tissues, (ii) PSTVd-sRNA-mediated cleavage is not necessarily associated with decreased accumulation of the targeted mRNA, and (iii) the mRNAs targeted likely differ depending on the PSTVd strain, tomato cultivar, and/or experimental conditions. In such a complex situation, it was not possible to identify which one, if any, of the targeted mRNAs is actually involved in the elicitation of the typical stunting and leaf curling symptoms observed in PSTVd infected tomato.

In the absence of biological replicates, selection of true targets in the *N. benthamiana* degradome was necessarily less precise, and, therefore, a higher number of possible PSTVd-sRNA targets was identified. Notably, none of these targets was a gene homologous to any of the eight bona fide tomato targets discussed, mainly because the mRNA sequences targeted by the PSTVd-sRNAs in tomato were not conserved in the homologous *N. benthamiana* genes (Table S6). The fact that both hosts exhibited similar stunting and leaf curling symptoms further supports the conclusion that the eight bona fide targets identified in tomato are likely not directly involved in the elicitation of these symptoms. However, we cannot exclude that they could play a role in plant–viroid interaction.

Sampling in our study was limited to the late stages of infection; thus, we cannot exclude the possibility that earlier targeting of one or more mRNAs by PSTVd-sRNAs could have triggered a signaling cascade leading to the later appearance of macroscopic symptoms. No evidence for such hypothetical targeted mRNA potentially eliciting pathogenesis was detected by our analyses, however. This is quite different from the situation with the RNA silencing-activated pathogenesis of the chloroplast replicating viroid PLMVd. In this case, vd-sRNA-mediated degradation of a specific host mRNA is always spatially and temporally associated with symptom expression, even at late stages [18,19].

Transcriptomic analyses have thoroughly documented the alteration by PSTVd (and other nuclear-replicating viroids) of the expression of genes involved in defense, hormone signaling, photosynthesis, RNA processing and binding, protein metabolism and modification, RNA silencing and plant innate immune responses [23,55,57,58,59,60]. The ability of viroids to interfere with the translational machinery [61,62], as well as with RNA-dependent DNA methylation [63,64,65,66,67] has also been reported. Besides the sequence-specific degradation of some host mRNA(s) guided by PSTVd-sRNAs, all these molecular pathways should be taken into consideration when PSTVd pathogenesis is addressed.

In summary, applying degradome analyses, we found several host mRNAs targeted by PSTVd-sRNAs for a specific cleavage mediated by RNA silencing machinery. However, based on comparative analysis in tomato and *N. benthamiana* plants, the direct involvement of these mRNAs in PSTVd-induced stunting and leaf curling symptoms in these hosts appears unlikely. Additional degradome-based studies performed at several infection stages could further clarify the role of RNA silencing in triggering PSTVd pathogenesis.

## 4. Materials and Methods

### 4.1. Plant Material

Seedlings of tomato (*Solanum lycopersicum* L.) cv. ‘Rutgers’ at the cotyledon/first true leaf stage were mechanically inoculated with nucleic acid extracts from tomato plants infected with PSTVd (Nb variant, GenBank accession number AJ634596.1) in 0.2 M boric acid-NaOH, pH 9 or with the inoculation buffer (mock-inoculated plants). *Nicotiana benthamiana* seedlings were agroinoculated with *Agrobacterium tumefaciens* C58 carrying either an empty plant binary expression plasmid (pBIN19Ø) or one containing a head-to-tail dimeric insert of PSTVd-Nb under the control of the 35S promoter of cauliflower mosaic virus [68]. Plants were grown in a controlled environment chamber at 28 °C with fluorescent light for 16 h and at 25 °C in darkness for 8 h. Leaf samples were collected 30 days post-inoculation (dpi). Three independent inoculation experiments were performed.

### 4.2. Total RNA Isolation and Northern Blot Hybridization

Total RNA was extracted from apical leaves of mock-inoculated and PSTVd-infected plants with TRizol Reagent (Invitrogen, Carlsbad, CA, USA) according to the manufacturer’s instructions. Total RNA preparations were analyzed by polyacrylamide gel electrophoresis under denaturing conditions (8M Urea and 1× TBE buffer) in 5% gels, for detecting genomic PSTVd RNAs, and in 17% gels, for detecting vd-sRNAs. After ethidium bromide staining, the RNAs were electrotransferred to Hybond-N+ membranes (Roche Diagnostics GmbH, Basel, Switzerland) and were hybridized with digoxigenin-labeled full-length riboprobes specific for detecting PSTVd of plus polarity as described previously [12]. Hybridization signals were revealed with anti-DIG alkaline phosphatase conjugate and the chemiluminescent substrate CSPD (Roche Diagnostics GmbH, Basel, Switzerland) and exposure to X-ray film. Ribosomal RNAs stained with ethidium bromide were used as equal loading controls.

### 4.3. Library Construction and High-Throughput Sequencing

cDNA libraries of sRNAs were generated as reported previously [26] and subjected to high-throughput sequencing on Hi-Seq 2500 (Illumina, San Diego, CA, USA) by Fasteris custom services (Fasteris, Geneva, Switzerland), generating 1 × 50 bp single-end reads.

Degradome libraries (Parallel Analysis of RNA Ends, PARE) were constructed according with the protocol described by German et al. [69]. Briefly, poly(A)+ RNA was isolated from 50 ug of total RNA obtained as indicated before. After ligation of an RNA adapter (Solexa Gex 1 adapter) to the 5′phosphate poly(A) + RNAs, the first cDNA strand was synthesized using an oligo-dT adapter primer, and the resulting cDNA was amplified by PCR. The amplicons were digested with *Mme*I and purified by PAGE before the Illumina 3′ TruSeq adapter was ligated to the *Mme*I overhang. After PCR amplification using a modified Gex1 adapter, the library products were sequenced (pair-end 2 × 25 bp) on Illumina NextSeq 500 system (Illumina, San Diego, CA, USA) by Vertis Biotechnologie AG (Freising, Germany). Raw sequences of the sRNAs and degradome libraries have been deposited in the European nucleotide archive (ENA) under the project accession number PRJEB42233.

### 4.4. Analysis of sRNA and Degradome Libraries

Raw sRNAs reads generated by HTS were filtered for quality and, after removing the adapters, they were sorted by sequence length. Reads of size between 18 to 26 nt were mapped by Bowtie 1.2.2 [70] on the PSTVd-Nb reference sequence and those of 21-nt length were selected for further degradome analysis. Tomato and *N. benthamiana* conserved miRNAs were identified by mapping (Bowtie 1.2.2) against the plant miRNA in miRbase (Release 22.1) and the published tomato and *N. benthamiana* miRNAs.

After quality control, adapter trimming, and filtering for 100% overlapping paired reads, high quality degradome reads were analyzed, using PAREsnip software tool [28] with a cut-off *p*-value of 0.05, for the identification of PSTVd-sRNA and miRNA targets. The transcripts data set used for mapping degradome reads was the release ITAGv2.3 of *Solanum lycopersicum*, and the v1.0.1 *N. benthamiana* GENOME predicted cDNA (www.solgenomics.net, accessed on 30 March 2021). The sRNA data set used in the PAREsnip tool to identify potential cleaved host targets of PSTVd-sRNAs in the degradome data from infected samples was constituted by the 21-nt sRNAs mapped in PSTVd obtained in the corresponding sRNA library (see above). Degradome data from mock controls was analyzed using a sRNA data set generated in silico to contain all possible sRNA derived from PSTVd-Nb. The resulting targets of PSTVd-sRNA in mock samples were considered as false targets.

The putative cleavage sites identified by PAREsnip analysis in each degradome library were assigned to one of five categories (categories 0–4) according to degradome fragment abundance at the cleaved position as described in [28]. This classification indicates the confidence level of target prediction, those in category 0 being the most likely to be true targets. In category 0, maximal abundance of the degradome tag occurs at the predicted cleavage site in the transcript. Category 1 is defined similarly, but there is more than one cleavage site that is associated with degradome tags of similar abundance. Category 2 sites are cleavage positions that are associated with tags whose abundance is lower than the maximum but higher than the median for that transcript. Category 3 sites are defined as for category 2 but where the abundance of tags at the predicted cleavage position is lower than or equal to the median. In category 4, degradome tag abundance may be as low as one raw read at that position. Abundance values for degradome tags in categories 0–3 are always more than one raw read.

### 4.5. RNA Ligase Mediate-Rapid Amplification of CDNA Ends (5′ RLM-RACE)

Aliquots (2 μg) of total RNA treated with turbo-DNase (Invitrogen, Carlsbad, CA, USA) were mixed with 0.25 μg of the RNA adapter (5′-CGACUGGAGCACGAGGACACUGACAUGGACUGAAGGAGUAGAAA-3′) and incubated at 95 °C for 2 min and snap-cooled on ice. Then, the mixture was incubated at 37 °C for one hour with 5U of T4 RNA ligase (Fermentas, Waltham, MA, USA), 1× buffer T4 RNA ligase (Fermentas, Waltham, MA, USA), 1 mM ATP and 10 U RNase inhibitor. First, cDNA synthesis of adapter-ligated RNAs was carried out with Superscript II (Invitrogen, Carlsbad, CA, USA) following the supplier instructions and using a specific primer complementary to the transcript of interest (Table S7). Then, the cDNA was amplified by PCR using the same primer of the first cDNA synthesis and a primer homologous to the RNA adapter (5ʹ-TGGAGCACGAGGACACTGACATG-3ʹ) and Go-Taq DNA polymerase (Promega, Corporation, Madison, WI, USA). PCR products of the expected size (Table S11) were eluted from agarose gel and re-amplified in a nested PCR reaction using a primer complementary to the transcript of interest and internal with respect to the primer used in the first PCR and a primer homologous to the RNA adapter and nested within the previous one (5ʹ-GACACTGACATGGACTGAAGGAGTAG-3ʹ). The PCR products were eluted from agarose gel, cloned in pGEMT-easy (Promega, Madison, WI, USA) and sequenced by Sanger custom sequencing service (Macrogen, Amsterdam, The Netherlands).

### 4.6. Quantitative RT-PCR (RT-qPCR) for Gene Expression Analysis

Total RNA extracted as described above was treated with Turbo DNase free (Invitrogene, Carlsbad, CA, USA) and 1 μg of the treated RNA was used for the first strand cDNA synthesis with Superscript II (Invitrogen, Carlsbad, CA, USA)) and random hexamers according to the manufacturer’s protocol. The cDNA reaction was diluted 5-fold with water and used as template for qPCR amplification. The PCR reaction was carried out in a total volume of 20 μL, containing: 4 μL of 5× PyroTaqEvaGreen qPCR Mix Plus (Bioline, London, UK), 0.5 μL of 10 μM of each primer, and 2 μL of the diluted cDNA reaction. Three biological replicates from three independent inoculation experiments were tested, and three technical replicates were performed for each biological replicate. Negative control reactions without cDNA template for each primer pair were included in each amplification experiment. The specificity of amplification was confirmed by melting curve analysis. Gene-specific primer pairs used for qPCR amplification are listed in Table S11. Efficiencies for each primer pair were calculated from standard curves obtained using serial dilutions of mock-inoculated tomato cDNA. To verify the sequence of the amplified cDNA, the amplicon generated in one of the RT-qPCR reactions for each pair of primers was cloned and sequenced. The relative expression level (fold change) of the selected transcripts was calculated by the 2^ΔΔCT^ method using the ubiquitin conjugating enzyme (UBC) as an internal reference gene for normalization of expression levels. Statistical significance was assessed by a *t*-test analysis, and the results were considered significant at *p* < 0.05. Error bars represent the standard error of the mean.

## Figures and Tables

**Table 1:** Bona fide mRNA targets of PSTVd-sRNA in tomato identified by degradome analysis.

mRNA Target	Degradome Results												PSTVd-sRNAs						
	A. Thaliana Homolog	Alignment Score	mef (Kcal/mol)	PSTVd-sRNA:mRNA Hybrid	Cat	Cleavage Position	*p*-Value T3	Number of Tags T3	Normalized Weighted Number of Tags T3	*p*-Value T4	Number of Tags T4	Normalized Weighted Number of Tags T4	5′Position	Polarity	PSTVd Variants *	Reads T3	RPM T3	Reads T4	RPM T4
Solyc09g083420.2.1	AT1G56290.1 (CWF19)	2.5	−37.4	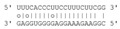	0	1587	0.00	74	1.93	0.00	149	2.62	178	(+)	Nb, Int, Mild, RG1, Lethal	935	25.22	1330	35.89
Solyc05g007450.2.1	AT1G26840.1 (ORC6)	3.0	−29.6	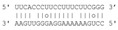	0	645	0.00	113	2.95	0.00	259	4.55	179	(+)	Nb, Int, mild, RG1, Lethal	2037	54.97	2931	79.1
Solyc11g012270.1.1	AT3G07880.1 (SCN1)	3.5	−31.0	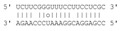	0	451	0.01	8	0.21	0.00	13	0.23	192	(+)	Nb, Int, RG1, Lethal	409	11.03	488	13.17
Solyc03g045050.2.1	AT1G67230.1 (LINC1)	3.5	−34.4	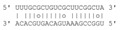	0	3520	0.00	196	5.12	0.00	316	5.55	238	(+)	Nb, Int, Mild, RG1, Lethal	2096	56.56	5535	149.4
Solyc05g024290.2.1	AT5G13160.1 (PBS1)	3.0	−29.6	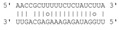	0	1542	0.00	56	1.46	0.01	163	2.86	290	(+)	Nb, Int, RG1, Lethal	138	3.72	542	14.63
Solyc12g007200.1.1	AT4G28980.1 (CDKF;1)	3.5	−33.7	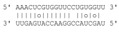	0	761	0.01	28	0.73	0.00	142	2.49	8	(+)	Nb, Int, mild, RG1, Lethal	277	7.47	354	9.553
Solyc04g079940.2.1	AT2G24430.1 (ANAC038)	3.0	−25.5	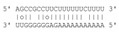	0	1339	0.00	33	0.86	0.02	38	0.67	70	(−)	Nb, Int, mild, RG1, Lethal	18	0.49	85	2.29
Solyc07g063100.2.1	AT4G27500.1 (PPI1)	3.0	−28.4	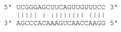	0	1675	0,00	637	16.65	0.00	2327	40.87	288	(−)	Nb, Int, mild, RG1, Lethal	319	8.61	458	12.36

* The sequence of the PSTVd-sRNA is conserved in the reported variants, ID of PSTVd variants: Nb (AJ634596), Int (AY937179); Mild (M14814.1); RG1 (U23058.1); Lethal (X58388.1).

## Data Availability

Raw sequences of the sRNAs and degradome libraries have been deposited in the European nucleotide archive (ENA) under the project accession number PRJEB42233.

## Supplementary Materials

The following are available online at https://www.mdpi.com/article/10.3390/ijms22073725/s1.

## References

[1] Flores R., Minoia S., Carbonell A., Gisel A., Delgado S., López-Carrasco A., Navarro B., Di Serio F. (2015). Viroids, the simplest RNA replicons: How they manipulate their hosts for being propagated and how their hosts react for containing the infection. Virus Res..

[2] Di Serio F., Owens R.A., Li S.F., Matoušek J., Pallás V., Randles J.W., Sano T., Verhoeven J.T.J., Vidalakis G., Flores R. (2020). ICTV Report Consortium. ICTV Virus Taxonomy Profile. Pospiviroidae. J. Gen. Virol..

[3] Di Serio F., Li S.F., Matoušek J., Owens R.A., Pallás V., Randles J.W., Sano T., Verhoeven J.T.J., Vidalakis G., Flores R. (2018). ICTV Report Consortium. ICTV Virus Taxonomy Profile: Avsunviroidae. J. Gen. Virol..

[4] Flores R., Navarro B., Delgado S., Serra P., Di Serio F. (2020). Viroid pathogenesis: A critical appraisal of the role of RNA silencing in triggering the initial molecular lesion. FEMS Microbiol. Rev..

[5] Adkar-Purushothama C.R., Perreault J.P. (2020). Current overview on viroid-host interactions. Wiley Interdiscip. Rev. RNA.

[6] Fukudome A., Fukuhara T. (2017). Plant dicer-like proteins: Double-stranded RNA-cleaving enzymes for small RNA biogenesis. J. Plant. Res..

[7] Ma Z., Zhang X. (2018). Actions of plant Argonautes: Predictable or unpredictable?. Curr. Opin. Plant Biol..

[8] Itaya A., Folimonov A., Matsuda Y., Nelson R.S., Ding B. (2001). Potato spindle tuber viroid as inducer of RNA silencing in infected tomato. Mol. Plant -Microbe Interact..

[9] Papaefthimiou I., Hamilton A.J., Denti M.A., Baulcombe D.C., Tsagris M., Tabler M. (2001). Replicating potato spindle tuber viroid RNA is accompanied by short RNA fragments that are characteristic of post-transcriptional gene silencing. Nucleic Acids Res..

[10] Martínez de Alba A.E., Flores R., Hernández C. (2002). Two chloroplastic viroids induce the accumulation of the small RNAs associated with post-transcriptional gene silencing. J. Virol..

[11] Wang M.B., Bian X.Y., Wu L.M., Liu L.X., Smith N.A., Isenegger D., Wu R.M., Masuta C., Vance V.B., Watson J.M. (2004). On the role of RNA silencing in the pathogenicity and evolution of viroids and viral satellites. Proc. Natl. Acad. Sci. USA.

[12] Minoia S., Carbonell A., Di Serio F., Gisel A., Carrington J.C., Navarro B., Flores R. (2014). Specific ARGONAUTES bind selectively small RNAs derived from potato spindle tuber viroid and attenuate viroid accumulation in vivo. J. Virol..

[13] Mi S., Cai T., Hu Y., Chen Y., Hodges E., Ni F., Wu L., Li S., Zhou H., Long C. (2008). Sorting of small RNAs into Arabidopsis argonaute complexes is directed by the 5′ terminal nucleotide. Cell.

[14] Montgomery T.A., Howell M.D., Cuperus J.T., Li D., Hansen J.E., Alexander A.L., Chapman E.J., Fahlgren N., Allen E., Carrington J.C. (2008). Specificity of ARGONAUTE7-miR390 interaction and dual functionality in TAS3 trans-acting siRNA formation. Cell.

[15] Hernández C., Flores R. (1992). Plus and minus RNAs of Peach latent mosaic viroid self-cleave in vitro via hammerhead structures. Proc. Natl. Acad. Sci. USA.

[16] Malfitano M., Di Serio F., Covelli L., Ragozzino A., Hernández C., Flores R. (2003). Peach latent mosaic viroid variants inducing peach calico (extreme chlorosis) contain a characteristic insertion that is responsible for this symptomatology. Virology.

[17] Rodio M.E., Delgado S., De Stradis A., Gómez M.D., Flores R., Di Serio F. (2007). A viroid RNA with a specific structural motif inhibits chloroplast development. Plant Cell.

[18] Delgado S., Navarro B., Serra P., Gentit P., Cambra M.A., Chiumenti M., De Stradis A., Di Serio F., Flores R. (2019). How sequence variants of a plastid-replicating viroid with one single nucleotide change initiate disease in its natural host. RNA Biol..

[19] Navarro B., Gisel A., Rodio M.E., Delgado S., Flores R., Di Serio F. (2012). Small RNAs containing the pathogenic determinant of a chloroplast-replicating viroid guide the degradation of a host mRNA as predicted by RNA silencing. Plant J..

[20] Adkar-Purushothama C.R., Brosseau C., Giguère T., Sano T., Moffett P., Perreault J.P. (2015). Small RNA derived from the virulence modulating region of the potato spindle tuber viroid silences callose synthase genes of tomato plants. Plant Cell.

[21] Adkar-Purushothama C.R., Iyer P.S., Perreault J.P. (2017). Potato spindle tuber viroid infection triggers degradation of chloride channel protein CLC-b-like and Ribosomal protein S3a-like mRNAs in tomato plants. Sci. Rep..

[22] Adkar-Purushothama C.R., Perreault J.P. (2018). Alterations of the viroid regions that interact with the host defense genes attenuate viroid infection in host plant. RNA Biol..

[23] Zheng Y., Wang Y., Ding B., Fei Z. (2017). Comprehensive transcriptome analyses reveal that potato spindle tuber viroid triggers genome-wide changes in alternative splicing, inducible trans-acting activity of phased secondary small interfering RNAs, and immune responses. J. Virol..

[24] Addo-Quaye C., Eshoo T.W., Bartel D.P., Axtell M.J. (2008). Endogenous siRNA and miRNA targets identified by sequencing of the Arabidopsis degradome. Curr. Biol..

[25] German M.A., Pillay M., Jeong D.H., Hetawal A., Luo S., Janardhanan P., Kannan V., Rymarquis L.A., Nobuta K., German R. (2008). Global identification of microRNA-target RNA pairs by parallel analysis of RNA ends. Nat. Biotechnol..

[26] Di Serio F., Martínez de Alba A.E., Navarro B., Gisel A., Flores R. (2010). RNA-dependent RNA polymerase 6 delays accumulation and precludes meristem invasion of a viroid that replicates in the nucleus. J. Virol..

[27] Adkar-Purushothama C.R., Perreault J.P., Sano T. (2015). Analysis of small RNA production patterns among the two potato spindle tuber viroid variants in tomato plants. Genom. Data.

[28] Folkes L., Moxon S., Woolfenden H.C., Stocks M.B., Szittya G., Dalmay T., Moulton V. (2012). PAREsnip: A tool for rapid genome-wide discovery of small RNA/target interactions evidenced through degradome sequencing. Nucleic Acids Res..

[29] Moxon S., Jing R., Szittya G., Schwach F., Rusholme Pilcher R.L., Moulton V., Dalmay T. (2008). Deep sequencing of tomato short RNAs identifies microRNAs targeting genes involved in fruit ripening. Genome Res..

[30] Itaya A., Bundschuh R., Archual A.J., Joung J.G., Fei Z., Dai X., Zhao P.X., Tang Y., Nelson R.S., Ding B. (2008). Small RNAs in tomato fruit and leaf development. Biochim. Biophys. Acta.

[31] Zhou R., Wang Q., Jiang F., Cao X., Sun M., Liu M., Wu Z. (2016). Identification of miRNAs and their targets in wild tomato at moderately and acutely elevated temperatures by high-throughput sequencing and degradome analysis. Sci. Rep..

[32] Karlova R., van Haarst J.C., Maliepaard C., van de Geest H., Bovy A.G., Lammers M., Angenent G.C., de Maagd R.A. (2013). Identification of microRNA targets in tomato fruit development using high-throughput sequencing and degradome analysis. J. Exp. Bot..

[33] Luan Y., Wang W., Liu P. (2014). Identification and functional analysis of novel and conserved microRNAs in tomato. Mol. Biol. Rep..

[34] Nuruzzaman M., Sharoni A.M., Satoh K., Karim M.R., Harikrishna J.A., Shimizu T., Sasaya T., Omura T., Haque M.A., Hasan S.M. (2015). NAC transcription factor family genes are differentially expressed in rice during infections with Rice dwarf virus, Rice black-streaked dwarf virus, Rice grassy stunt virus, Rice ragged stunt virus, and Rice transitory yellowing virus. Front. Plant Sci..

[35] Mathew I.E., Agarwal P. (2018). May the Fittest Protein Evolve: Favoring the Plant-Specific Origin and Expansion of NAC Transcription Factors. Bioessays.

[36] Jarad M., Mariappan K., Almeida-Trapp M., Mette M.F., Mithöfer A., Rayapuram N., Hirt H. (2020). The Lamin-Like LITTLE NUCLEI 1 (LINC1) Regulates Pattern-Triggered Immunity and Jasmonic Acid Signaling. Front. Plant Sci..

[37] Sakamoto Y., Takagi S. (2013). LITTLE NUCLEI 1 and 4 regulate nuclear morphology in Arabidopsis thaliana. Plant Cell Physiol..

[38] Bell S.P. (2002). The origin recognition complex: From simple origins to complex functions. Genes Dev..

[39] Collinge M.A., Spillane C., Köhler C., Gheyselinck J., Grossniklaus U. (2004). Genetic interaction of an origin recognition complex subunit and the Polycomb group gene MEDEA during seed development. Plant Cell.

[40] Qin Z., Zhang X., Zhang X., Xin W., Li J., Hu Y. (2014). The Arabidopsis transcription factor IIB-related protein BRP4 is involved in the regulation of mitotic cell-cycle progression during male gametogenesis. J. Exp. Bot..

[41] Carol R.J., Takeda S., Linstead P., Durrant M.C., Kakesova H., Derbyshire P., Drea S., Zarsky V., Dolan L. (2005). A RhoGDP dissociation inhibitor spatially regulates growth in root hair cells. Nature.

[42] Shimotohno A., Umeda-Hara C., Bisova K., Uchimiya H., Umeda M. (2004). The plant-specific kinase CDKF;1 is involved in activating phosphorylation of cyclin-dependent kinase-activating kinases in Arabidopsis. Plant Cell.

[43] Takatsuka H., Ohno R., Umeda M. (2009). The Arabidopsis cyclin-dependent kinase-activating kinase CDKF;1 is a major regulator of cell proliferation and cell expansion but is dispensable for CDKA activation. Plant J..

[44] Swiderski M.R., Innes R.W. (2001). The Arabidopsis PBS1 resistance gene encodes a member of a novel protein kinase subfamily. Plant J..

[45] Morandini P., Valera M., Albumi C., Bonza M.C., Giacometti S., Ravera G., Murgia I., Soave C., De Michelis M.I. (2002). A novel interaction partner for the C-terminus of Arabidopsis thaliana plasma membrane H^+^-ATPase (AHA1 isoform): Site and mechanism of action on H^+^-ATPase activity differ from those of 14-3-3 proteins. Plant J..

[46] Viotti C., Luoni L., Morandini P., De Michelis M.I. (2005). Characterization of the interaction between the plasma membrane H-ATPase of Arabidopsis thaliana and a novel interactor (PPI1). FEBS J..

[47] Baksa I., Nagy T., Barta E., Havelda Z., Várallyay É., Silhavy D., Burgyán J., Szittya G. (2015). Identification of *Nicotiana benthamiana* microRNAs and their targets using high throughput sequencing and degradome analysis. BMC Genom..

[48] Diermann N., Matousek J., Junge M., Riesner D., Steger G. (2010). Characterization of plant miRNAs and small RNAs derived from potato spindle tuber viroid (PSTVd) in infected tomato. Biol. Chem..

[49] Tsushima D., Adkar-Purushothama C.R., Taneda A., Sano T. (2015). Changes in relative expression levels of viroid-specific small RNAs and microRNAs in tomato plants infected with severe and mild symptom-inducing isolates of potato spindle tuber viroid. J. Gen. Plant Pathol..

[50] Owens R.A., Tech K.B., Shao J.Y., Sano T., Baker C.J. (2012). Global analysis of tomato gene expression during potato spindle tuber viroid infection reveals a complex array of changes affecting hormone Signaling. Mol. Plant Microbe Int..

[51] Martín R., Arenas C., Daròs J.A., Covarrubias A., Reyes J., Chua N. (2007). Characterization of small RNAs derived from Citrus exocortis viroid (CEVd) in infected tomato plants. Virology.

[52] Morel J.B., Godon C., Mourrain P., Béclin C., Boutet S., Feuerbach F., Proux F., Vaucheret H. (2002). Fertile hypomorphic ARGONAUTE (ago1) mutants impaired in post-transcriptional gene silencing and virus resistance. Plant Cell.

[53] Baumberger N., Baulcombe D.C. (2005). Arabidopsis ARGONAUTE1 is an RNA Slicer that selectively recruits microRNAs and short interfering RNAs. Proc. Natl. Acad. Sci. USA.

[54] Carbonell A., Carrington J.C. (2015). Antiviral roles of plant ARGONAUTES. Curr. Opin. Plant Biol..

[55] Góra-Sochacka A., Więsyk A., Fogtman A., Lirski M., Zagórski-Ostoja W. (2019). Root Transcriptomic Analysis Reveals Global Changes Induced by Systemic Infection of Solanum lycopersicum with Mild and Severe Variants of Potato Spindle Tuber Viroid. Viruses.

[56] Adkar-Purushothama C.R., Sano T., Perreault J.P. (2018). Viroid-derived small RNA induces early flowering in tomato plants by RNA silencing. Mol. Plant Pathol..

[57] Thibaut O., Bragard C. (2018). Innate immunity activation and RNAi interplay in citrus exocortis viroid—Tomato pathosystem. Viruses.

[58] Štajner N., Radišek S., Mishra A.K., Nath V.S., Matoušek J., Jakše J. (2019). Evaluation of disease severity and global transcriptome response induced by citrus bark cracking viroid, hop latent viroid, and their co-infection in hop (*Humulus lupulus* L.). Int. J. Mol. Sci..

[59] Takino H., Kitajima S., Hirano S., Oka M., Matsuura T., Ikeda Y., Kojima M., Takebayashi Y., Sakakibara H., Mino M. (2019). Global transcriptome analyses reveal that infection with chrysanthemum stunt viroid (CSVd) affects gene expression profile of chrysanthemum plants, but the genes involved in plant hormone metabolism and signaling may not be silencing target of CSVd-siRNAs. Plant Gene.

[60] Wang Y., Wu J., Qiu Y., Atta S., Zhou C., Cao M. (2019). Global Transcriptomic Analysis Reveals Insights into the Response of ‘Etrog’ Citron (*Citrus medica* L.) to Citrus Exocortis Viroid Infection. Viruses.

[61] Lisón P., Tárraga S., López-Gresa P., Saurí A., Torres C., Campos L., Bellés J.M., Conejero V., Rodrigo I. (2013). A noncoding plant pathogen provokes both transcriptional and posttranscriptional alterations in tomato. Proteomics.

[62] Cottilli P., Belda-Palazón B., Adkar-Purushothama C.R., Perreault J.P., Schleiff E., Rodrigo I., Ferrando A., Lisón P. (2019). Citrus exocortis viroid causes ribosomal stress in tomato plants. Nucleic Acids Res..

[63] Castellano M., Martinez G., Pallás V., Gómez G. (2015). Alterations in host DNA methylation in response to constitutive expression of Hop stunt viroid RNA in Nicotiana benthamiana plants. Plant Pathol..

[64] Castellano M., Pallás V., Gómez G. (2016). A pathogenic long noncoding RNA redesigns the epigenetic landscape of the infected cells by subverting host Histone Deacetylase 6 activity. New Phytol..

[65] Lv D.Q., Liu S.W., Zhao J.H., Zhou B.J., Wang S.P., Guo H.S., Fang Y.Y. (2016). Replication of a pathogenic non-coding RNA increases DNA methylation in plants associated with a bromodomain-containing viroid-binding protein. Sci. Rep..

[66] Martinez G., Castellano M., Tortosa M., Pallás V., Gómez G. (2014). A pathogenic non-coding RNA induces changes in dynamic DNA methylation of ribosomal RNA genes in host plants. Nucleic Acids Res..

[67] Torchetti E.M., Pegoraro M., Navarro B., Catoni M., Di Serio F., Noris E. (2016). A nuclear-replicating viroid antagonizes infectivity and accumulation of a geminivirus by upregulating methylation-related genes and inducing hypermethylation of viral DNA. Sci. Rep..

[68] Carbonell A., Flores R., Gago S. (2011). Trans-cleaving hammerhead ribozymes with tertiary stabilizing motifs: In vitro and in vivo activity against a structured viroid RNA. Nucleic Acids Res..

[69] German M.A., Luo S., Schroth G., Meyers C., Green P.J. (2009). Construction of Parallel Analysis of RNA Ends (PARE) libraries for the study of cleaved miRNA targets and the RNA degradome. Nat. Protocol..

[70] Langmead B., Trapnell C., Pop M., Salzberg S.L. (2009). Ultrafast and memory-efficient alignment of short DNA sequences to the human genome. Genome Biol..

