# Supplementary material for: Degradome Analysis of Tomato and Nicotiana benthamiana Plants Infected with Potato Spindle Tuber Viroid
Source: Int J Mol Sci. 2021 Apr 2;22(7):3725. doi: 10.3390/ijms22073725 (PMC8038209; doi:10.3390/ijms22073725)
Supplement: Supplementary file 1 [file ijms-22-03725-s001.zip › Supplementary material/Supplementary figures S1 to S6.pdf]

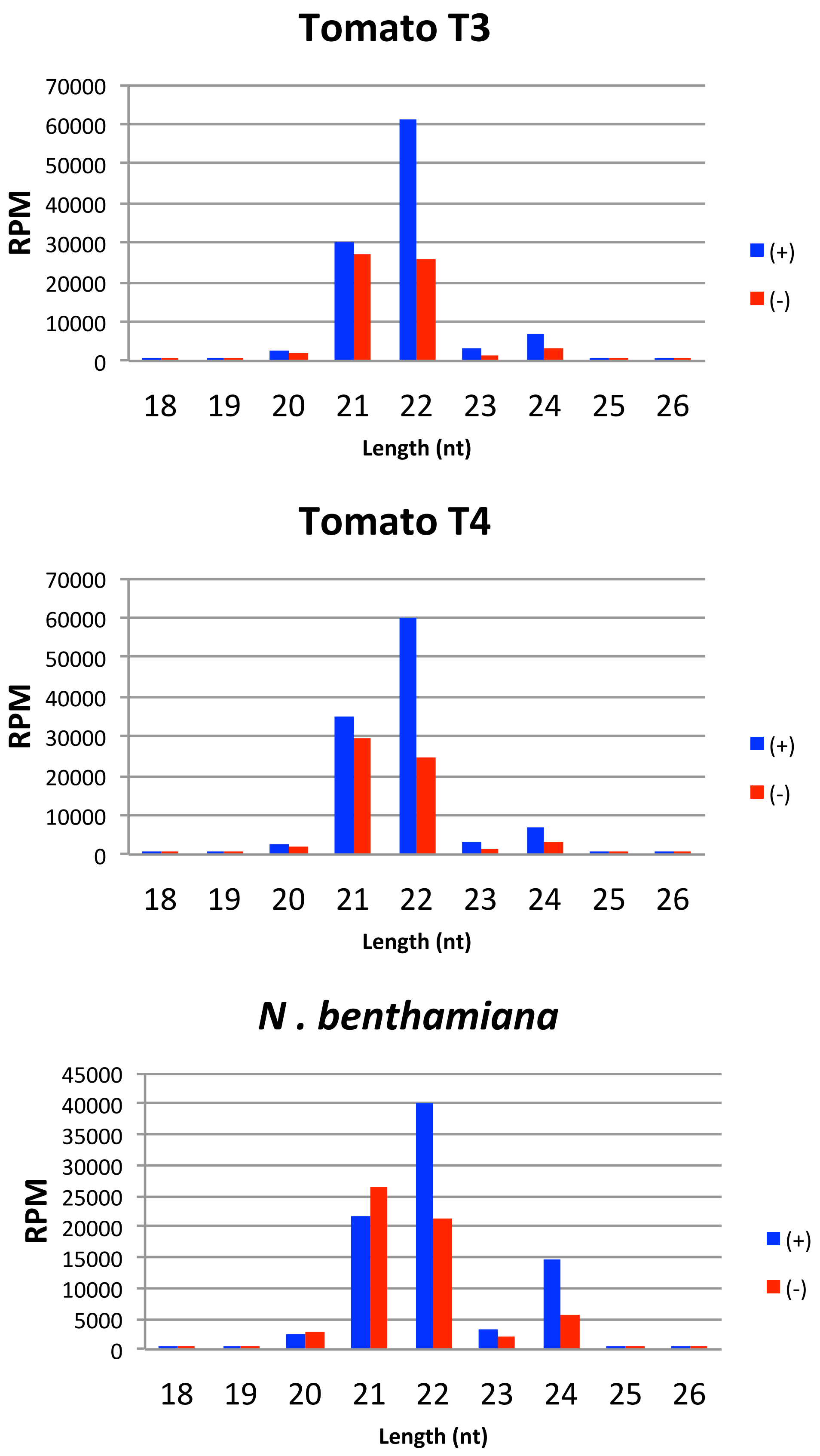

Figure S1. Size and strand polarity distribution of PSTVd-sRNA (18 to 26 nt) derived from the tomato (T3 and T4) and *N. benthamiana* PSTVd-infected samples. RPM, reads per million.

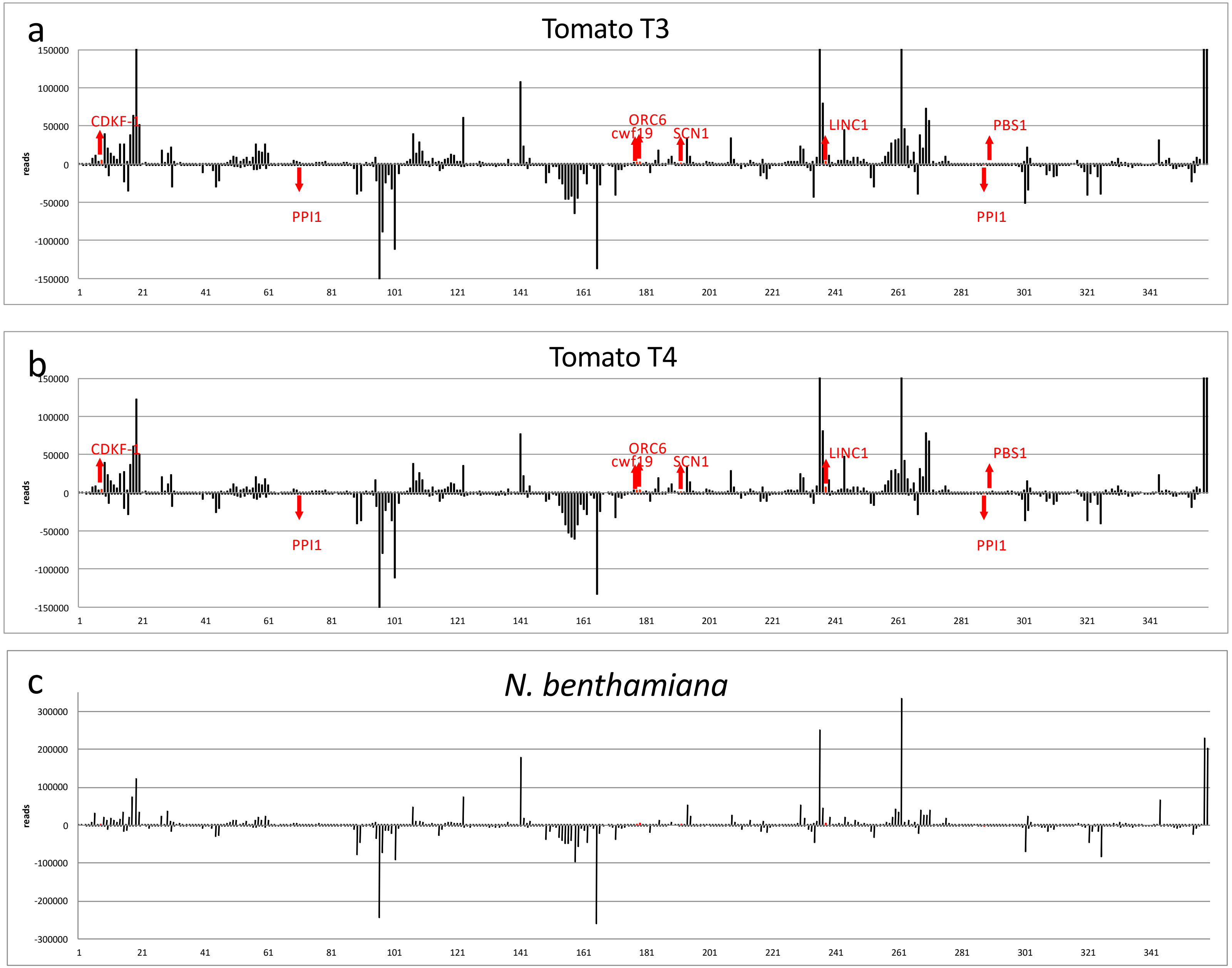

Figure S2. Distribution of PSTVd-sRNAs along PSTVd genome. Location and frequencies in the genomic PSTVd RNA (variant Nb) of the 5′-terminal nucleotide of the plus-strand (positive values) and minus-strand (negative values) PSTVd-sRNAs from the viroid-infected tomato T3 and T4 (**a** and **b**, respectively) and *N. benthamiana* (**c**) plants. Red arrows indicate the PSTVd-sRNAs identified in the degradome analysis that mediate the cleavage of a *bona-fide* mRNA target (the name of which is in red).

Mock (T1) | Mock (T2) | PSTVd-infected (T3) | PSTVd-infected (T4)

Solyc03g114850.2.1 (Squamosa promoter-binding protein-like) CLEAVAGE POSITION 1481 Targeted by mir156

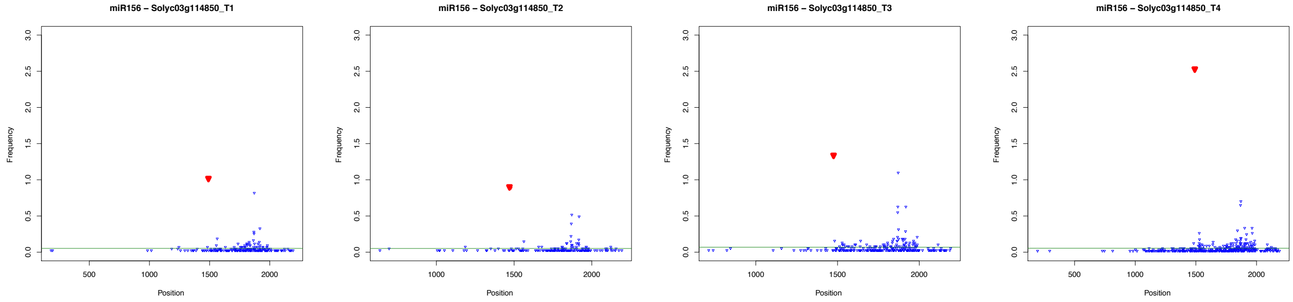

Solyc05g015840.2.1 (Squamosa promoter-binding protein-like) CLEAVAGE POSITION 760; Targeted by mir156

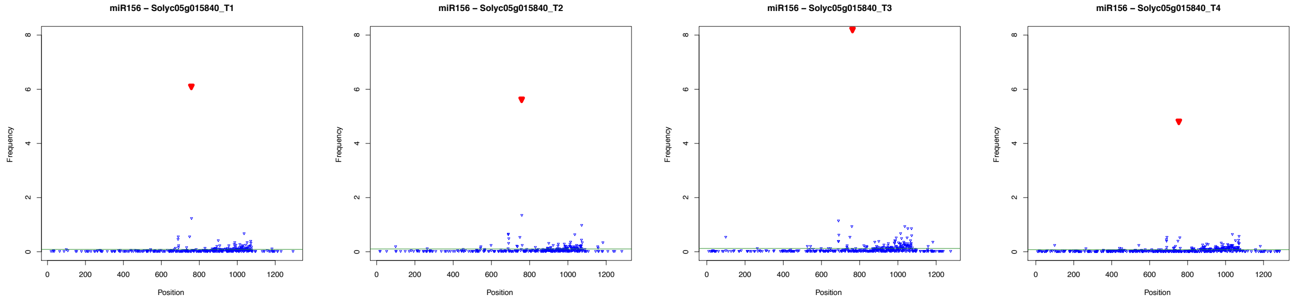

Solyc02g037530.2.1 (auxin response factor 8) CLEAVAGE POSITION 2291; targeted by miR167

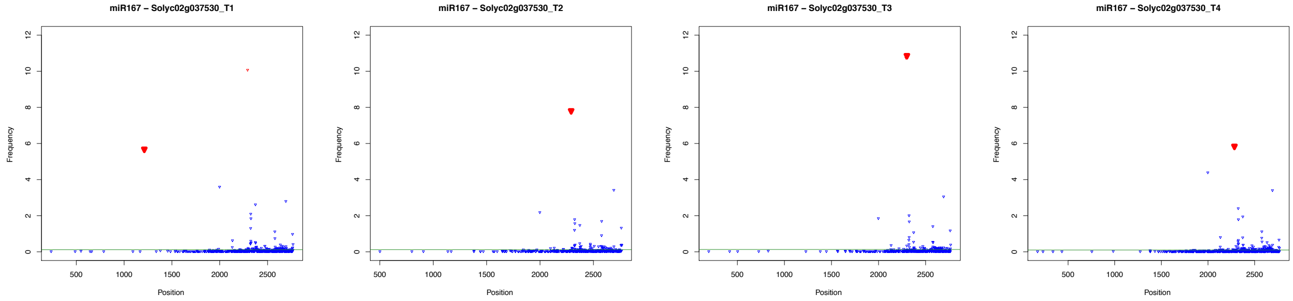

Solyc01g087240.2.1 (nuclear factor Y, subunit A9) CLEAVAGE POSITION 1171 ; targeted by miR169

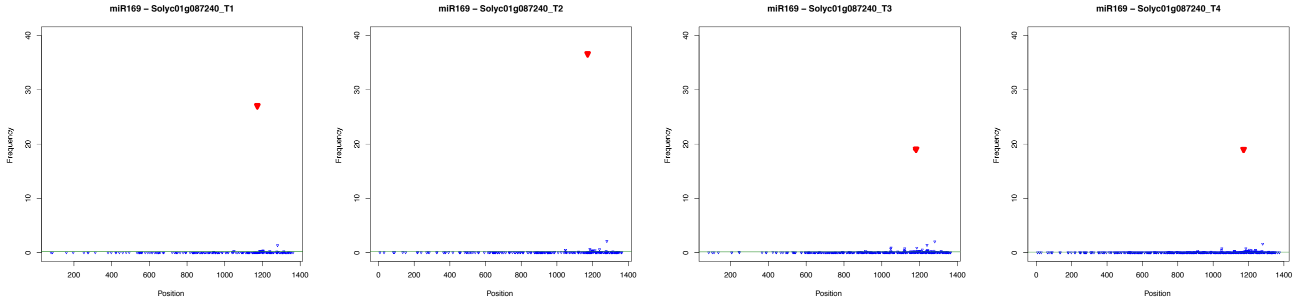

Solyc11g072600.1.1 (related to AP2.7) CLEAVAGE POSITION 1288 ; targeted by miR172

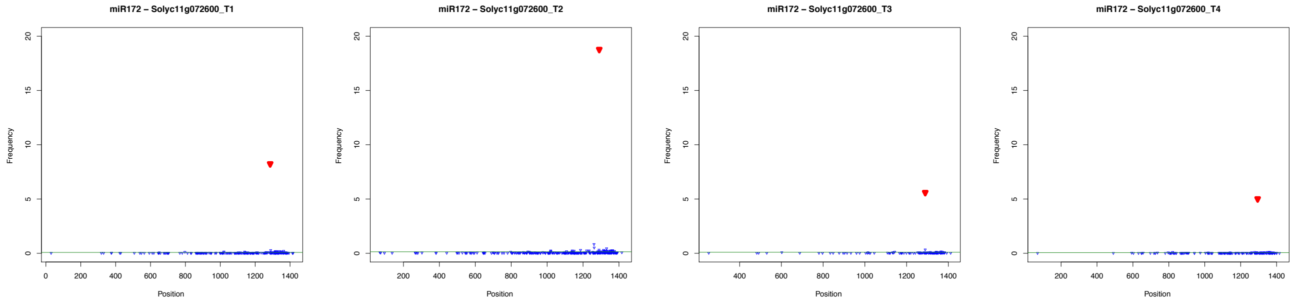

Mock (T1)	Mock (T2)	PSTVd-infected (T3)	PSTVd-infected (T4)

Solyc12g014140.1.1 (TCP family transcription factor 4) CLEAVAGE POSITION 917 ; targeted by miR319

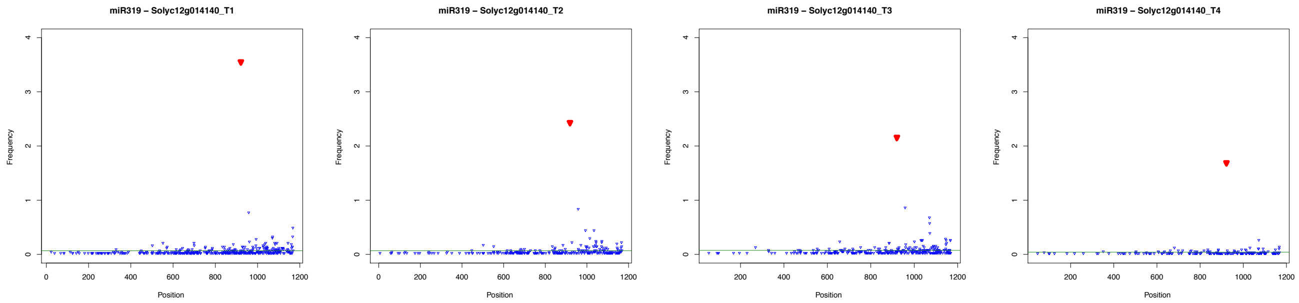

Solyc08g083230.1.1 (growth-regulating factor 8) CLEAVAGE POSITION 491; targeted by miR396

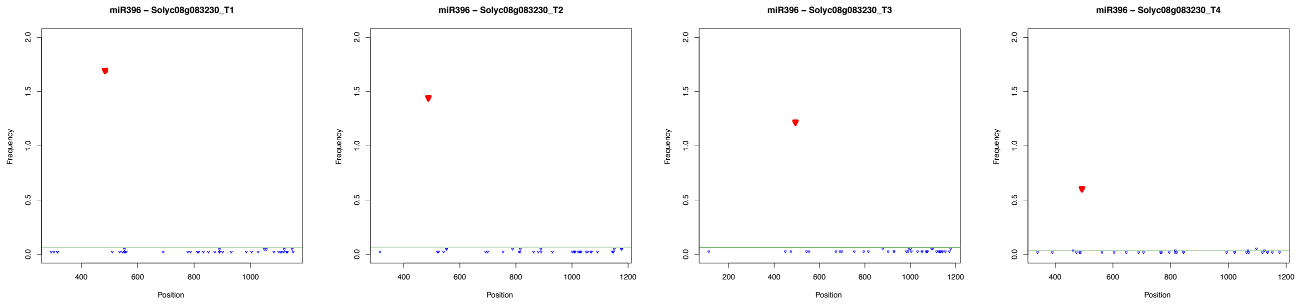

Solyc01g067740.2.1 (copper/zinc superoxide dismutase 1. CSD1) CLEAVAGE POSITION 75; targeted by miR398

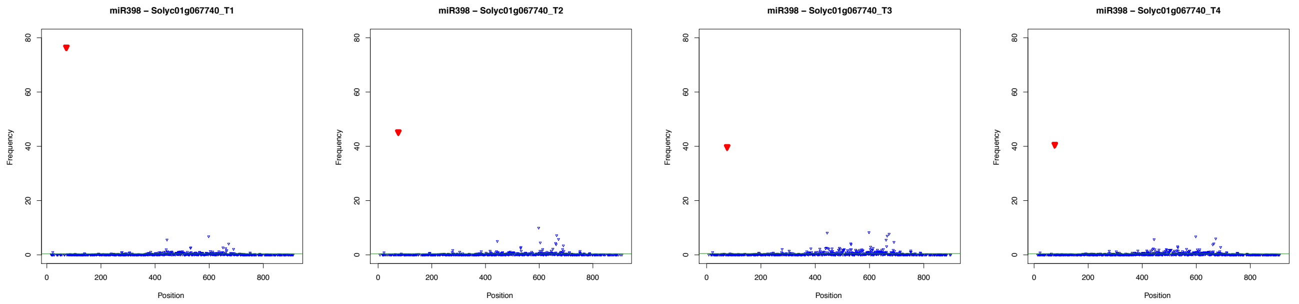

Solyc01g104400.2.1 (Plantacyanin) CLEAVAGE POSITION 88; targeted by miR408

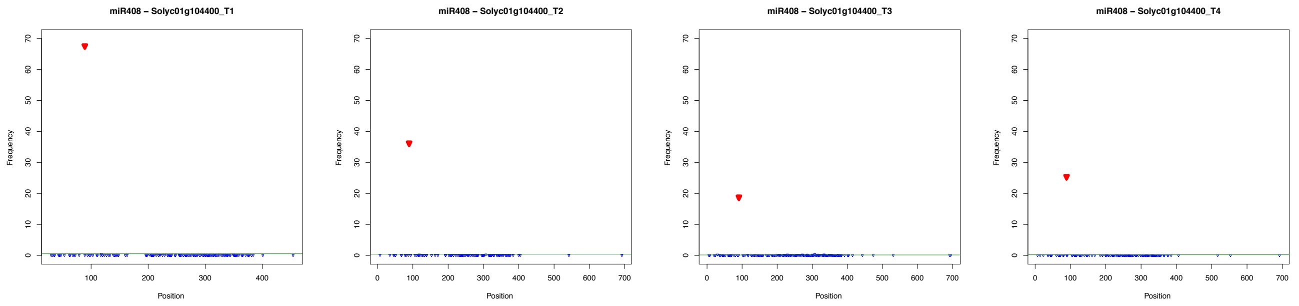

Figure S3. T-plots of mRNA targets of representative conserved miRNAs in mock (T1 and T2) and PSTVd-infected (T3 and T4) replicates. The abundance of each degradome tag is plotted as a function of the position of its 5′ end in the transcript. In each t-plot, the tag abundance at the cleavage site predicted according with the cognate miRNA is reported in red.

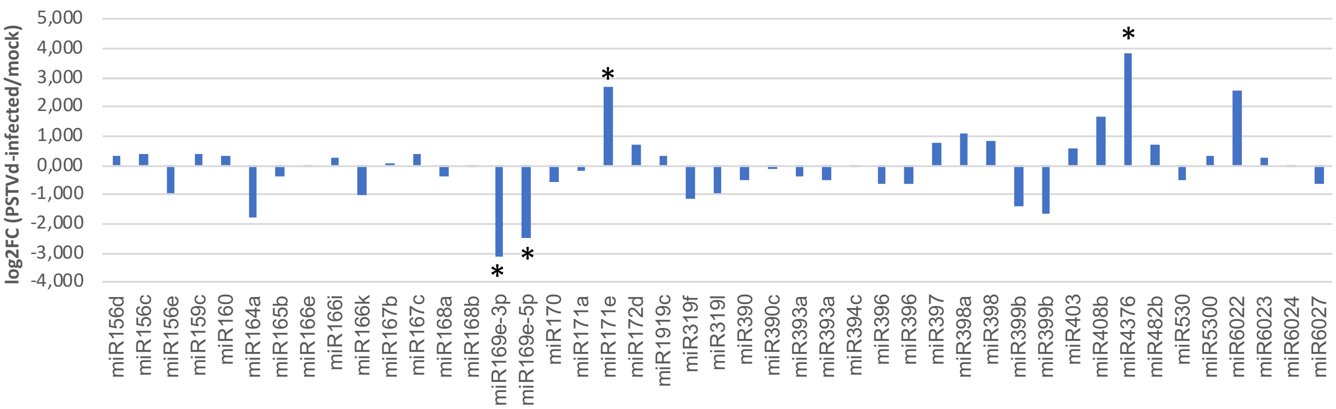

Figure S4. Changes in accumulation levels of miRNAs upon PSTVd-infection. log_2 of the fold change (FC) of miRNAs accumulation in PSTVd-infected with respect to mock-inoculated tomato plant are shown. The read per million average in the two replicates was used to calculate the FC. Statistical significance for t-test ($P < 0.01$) is indicated by asterisks.

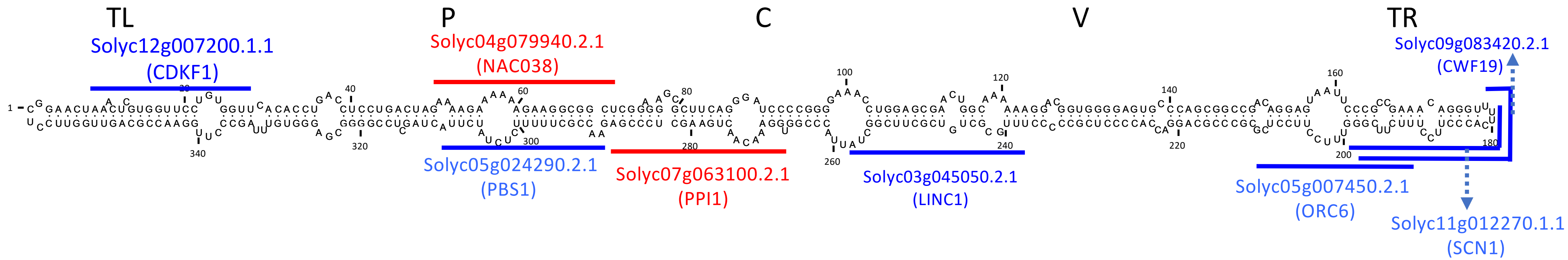

Figure S5. Localization in the PSTVd proposed secondary structure of the PSTVd-sRNAs targeting for cleavage eight *bona fide* tomato mRNAs. Blue and red lines denote the plus and minus polarity strand of the PSTVd-sRNAs, respectively. The ID and the name of the mRNAs targeted by each PSTVd-sRNA are indicated.

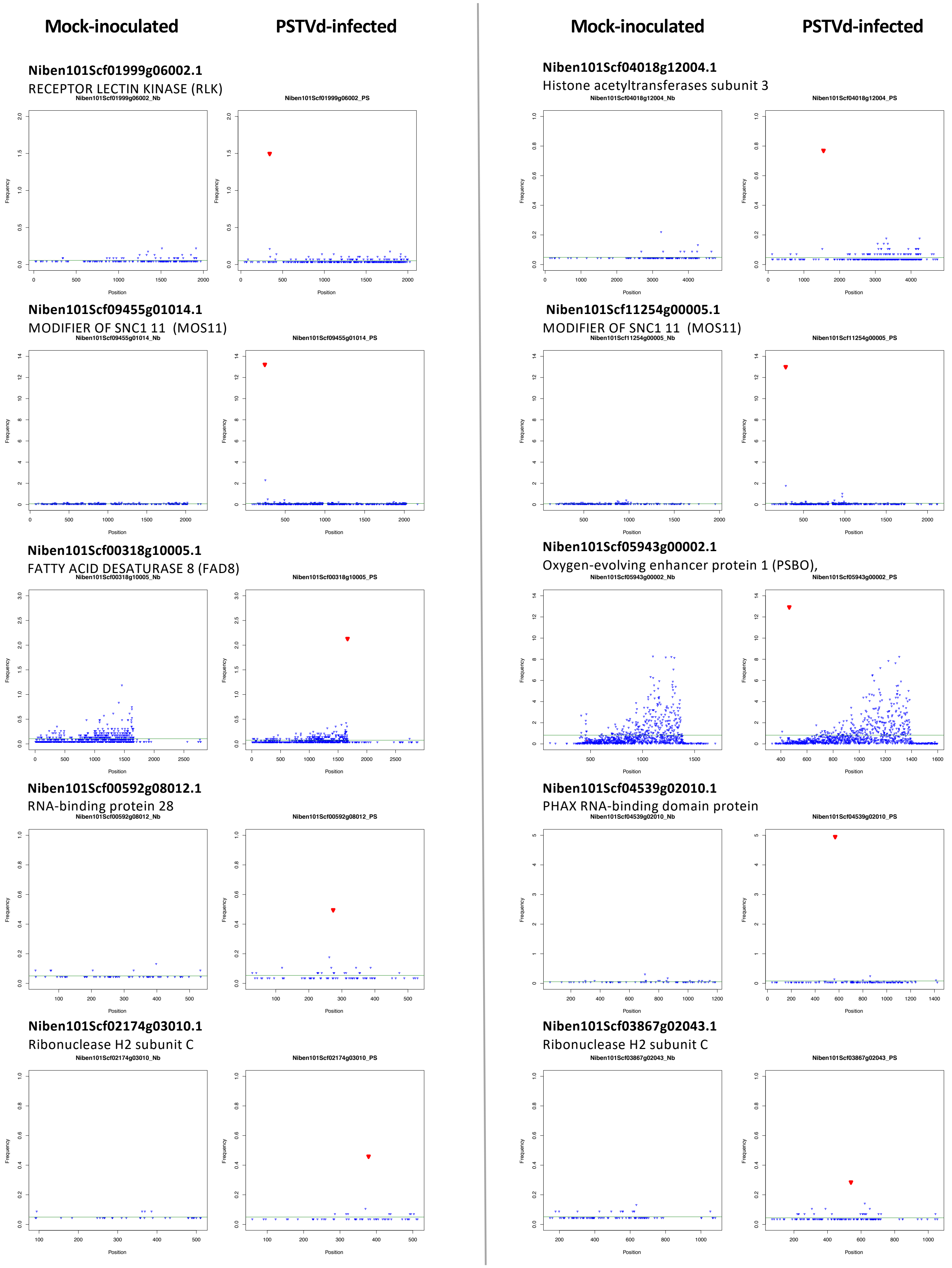

Figure S6. T-plots of ten representative specific mRNA targets of PSTVd-sRNA identified in mock-inoculated and PSTVd-infected inoculated *N. benthamiana*. The abundance of each degradome tag is plotted as a function of the position of its 5′ end in the transcript. In each t-plot from PSTVd-infected sample, the tag abundance at the cleavage site predicted according with the cognate PSTVd-sRNA is reported in red. The ID and the name of the mRNAs targeted by each PSTVd-sRNA are indicated.
